# Increasing Incidence of *Salmonella* in Australia, 2000-2013

**DOI:** 10.1371/journal.pone.0163989

**Published:** 2016-10-12

**Authors:** Laura Ford, Kathryn Glass, Mark Veitch, Rebecca Wardell, Ben Polkinghorne, Timothy Dobbins, Aparna Lal, Martyn D. Kirk

**Affiliations:** 1 National Centre for Epidemiology and Population Health, The Australian National University, Canberra, Australian Capital Territory (ACT), Australia; 2 Department of Health and Human Services, Hobart, Tasmania (Tas.), Australia; 3 OzFoodNet, Australian Government Department of Health, Canberra, Australian Capital Territory (ACT), Australia; Indian Institute of Science, INDIA

## Abstract

*Salmonella* is a key cause of foodborne gastroenteritis in Australia and case numbers are increasing. We used negative binomial regression to analyze national surveillance data for 2000–2013, for *Salmonella* Typhimurium and non-Typhimurium *Salmonella* serovars. We estimated incidence rate ratios adjusted for sex and age to show trends over time. Almost all states and territories had significantly increasing trends of reported infection for *S*. Typhimurium, with states and territories reporting annual increases as high as 12% (95% confidence interval 10–14%) for *S*. Typhimurium in the Australian Capital Territory and 6% (95% CI 5–7%) for non-Typhimurium *Salmonella* in Victoria. *S*. Typhimurium notification rates were higher than non-Typhimurium *Salmonella* rates in most age groups in the south eastern states of Australia, while non-Typhimurium rates were higher in most age groups elsewhere. The *S*. Typhimurium notification rate peaked at 12–23 months of age and the non-Typhimurium *Salmonella* notification rate peaked at 0–11 months of age. The age-specific pattern of *S*. Typhimurium cases suggests a foodborne origin, while the age and geographic pattern for non-Typhimurium may indicate that other transmission routes play a key role for these serovars.

## Introduction

*Salmonella enterica* is transmitted via food, the environment, water, people and animals, and often causes gastroenteritis in humans [1, 2]. Worldwide, *Salmonella* infections, excluding those caused by *S*. Typhi and *S*. Paratyphi, were estimated in a paper published in 2010 to cause 93.8 million (90% credible interval 61.8–131.6 million) cases of gastroenteritis per year, 80.3 million of which are considered foodborne [3]. Approximately 72% of salmonellosis in Australia is estimated to be transmitted through contaminated food [1]. Common foods associated with salmonellosis in outbreak investigations and source attribution studies include eggs, poultry meat, pork, beef, dairy products, nuts, and fresh produce [4, 5, 6, 7].

There are over 2,500 different serovars of *Salmonella* [8]. The most common serovar in Australia is *S*. Typhimurium, which is also the most commonly identified etiological agent in outbreaks [7, 9]. *S*. Enteritidis is not endemic in Australian poultry layer flocks and most human infections with *S*. Enteritidis are acquired overseas [7]. Many other serovars occupy distinct ecological niches and epidemiological foci in Australia, as it is a large country with wide climatic and geo-physical variation [7,10].

In Australia, all laboratory confirmed *Salmonella* infections are reported to state and territory health departments, and subsequently to the National Notifiable Diseases Surveillance System (NNDSS) [11]. Surveillance data are an under representation of the total burden of salmonellosis cases, with an estimated 7 salmonellosis cases (95% CI 4–16) occurring in the community for every 1 notification to health departments [12]. Circa 2010, there were an estimated 40,000 salmonellosis cases attributable to contaminated food in the Australian community each year [13]. In addition, there were an estimated 2,100 hospitalizations, 15 deaths and 6,750 complications from contaminated food [13,14].

National surveillance figures over the last decade suggest the rate of *Salmonella* infections has been increasing [15]. We compared the rates of *Salmonella* infections between sex, age groups, and Australian states and territories over 2000–2013 in order to examine trends in the reported incidence of infection and identify differences between states and territories.

## Materials and Methods

**Fig 1:**
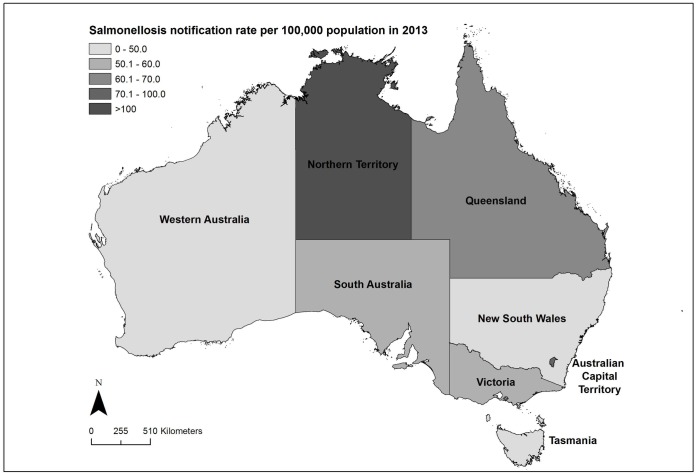
Map of Australian States and Territories showing the crude notification rate of salmonellosis for 2013 after excluding cases with missing data on serovar, age, or sex. Administrative boundaries from the Australian Bureau of Statistics [16].

In this study, we used national Australian human salmonellosis notification data to analyze disease trends by state and territory from 2000 to 2013. In Australia, there are six states: New South Wales (NSW), Queensland (Qld), South Australia (SA), Tasmania (Tas.), Victoria (Vic.), and Western Australia (WA); and two territories: the Australian Capital Territory (ACT) and the Northern Territory (NT) (Fig 1). The climate and environment varies widely both across and within these states and territories. We chose to examine *Salmonella* trends at a state and territory level due to the availability of data at that level, and evidence that suggests that the frequency of *Salmonella* serovars differs by state and territory [7].

Ethics approval for this study was granted by the Australian National University Human Research Ethics Committee [protocol 2012/412].

### Data sources

All states and territories have public health legislation that requires doctors and/or pathology laboratories to report any confirmed cases of salmonellosis [17]. A confirmed case requires definitive laboratory evidence of the isolation or detection of *Salmonella* species, excluding *S*. Typhi, which is notified separately [18]. State-based surveillance systems have collected data and entered all confirmed salmonellosis cases into NNDSS since 1991. We requested de-identified *Salmonella* spp. NNDSS notifications (including ‘notification receive date’, ‘true onset date’, ‘diagnosis date’, ‘age at onset’, ‘sex’, ‘organism’, and ‘serogroup’ fields) for each state and territory for 1991 to 2013 from the Communicable Disease Network of Australia (CDNA). We used ‘diagnosis date’ for all analyses, which is defined as the date a person’s illness began (onset), or where onset date is unknown, the earliest of the specimen collection date, the date the health professional signed the notification form or the laboratory issued the results, or the date the notification is received by the communicable diseases section of the health authority. Due to incompleteness of serotyping data in the NNDSS in the 1990s, we restricted the data for analysis to cases with diagnosis dates from 2000 to 2013 inclusive.

Rates of illness per 100,000 population were calculated using the estimated resident population by age and sex for each state and territory as of the June quarter for each year between 2000 and 2013 from the Australian Bureau of Statistics (ABS) [19].

### Analysis

The primary aim of the analysis was to examine the trends in reported incidence over the time period. We excluded all *Salmonella* cases where the serovar, age, or sex was missing. We also excluded infections due to *S*. Paratyphi A, *S*. Paratyphi B (except for bioser Java), and *S*. Paratyphi C, as they predominantly result in enteric fever and are acquired while traveling overseas. *S*. Typhimurium and non-Typhimurium *Salmonella* were analyzed separately to examine the trends of Australia’s most common serovar, *S*. Typhimurium, compared with trends of all other types of non-typhoidal *Salmonella* notified in Australia. Less than 0.1% of notifications were typed as *S*. subspecies *enterica*, which were grouped with *S*. Typhimurium if they had an H = i in the antigenic formula or a known Typhimurium phage type (commonly known as monophasic *S*. Typhimurium). We used negative binomial regression to estimate incidence rate ratios (IRR) by sex, age, and state and territory. We also included an interaction term in the model to estimate IRRs for trend over time by state and territory. ABS population numbers by age, sex and state and territory were used as an offset to standardize the incidence rates to the population. We used the interaction between state/territory and year to produce a trend over time for each state and territory, with year defined as the year of diagnosis and treated as a continuous variable. A state-by-state analysis showed that treating year as a continuous variable was appropriate for states and territory data (NT excepted—see S1 Fig). Age was categorized in one-year age groups from 0 to 4 and in 5-year age groups until 85 years and over. Analysis was performed with Stata statistical package 12.1 (www.stata.com/) and graphs were made using Stata and Microsoft Excel 2010. ArcGIS v10.3 (http://www.esri.com/software/arcgis) was used to create a map of the crude annual salmonellosis notification rate in 2013.

### Sensitivity analysis

NNDSS data does not allow us to distinguish between sporadic and outbreak cases. To test the effect of years with large outbreaks or a large number of sporadic cases on our rate ratio estimates, we removed outlier years of 2009 in ACT, 2011 in SA, and 2005 in Tas. from the *S*. Typhimurium analysis. An outlier year was defined as any year where there was an absolute difference of 10 per 100,000 or greater between the crude rate and the predicted rate.

## Results

There were 127,195 cases of salmonellosis reported to NNDSS with a diagnosis date between 2000 and 2013. Of these notifications, 97.7% (124,235/127,195) included serovar data (S1 Table) and of those, 99.6% (123,762/124,235) included age and sex data. Nationally, the crude annual rate was lowest in 2000 (30.6 per 100,000) and increased to 53.0 per 100,000 in 2013 (S2 Table). Fig 1 shows the geography of the Australian states and territories, together with the crude notification rate of salmonellosis for 2013 by state.

**Fig 2:**
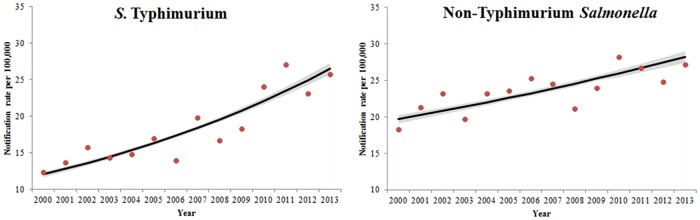
Crude notification rates (dots) and negative binomial regression margins plot (lines with 95% CI) of *S*. Typhimurium and non-Typhimurium *Salmonella* notification rates, Australia 2000–2013.

Rates of both *S*. Typhimurium and non-Typhimurium *Salmonella* increased from 2000 to 2013 with an IRR of 1.06 (95% CI 1.06–1.07) for *S*. Typhimurium and 1.03 (95% CI 1.02–1.03) for non-Typhimurium *Salmonella* (Fig 2). *S*. Typhimurium was the most frequently reported serovar and was responsible for 43.9% (54,313/123,762) of notifications over the time period. *S*. Enteritidis was responsible for 5.7% (7,001/123,762) of notifications. The proportion of notifications of *S*. Typhimurium and common non-Typhimurium serovars varied by state and territory (S3 Table).

**Fig 3:**
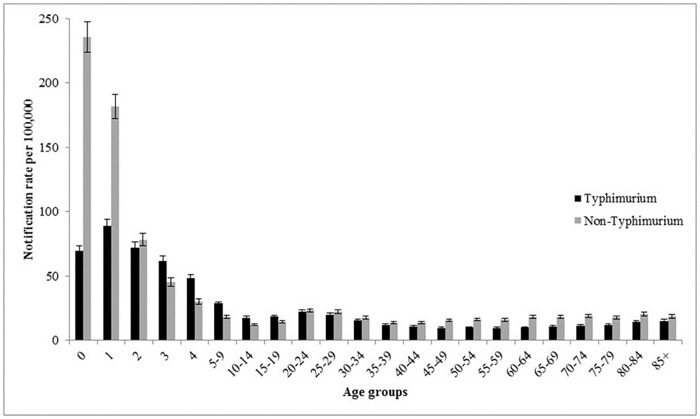
*Salmonella* Typhimurium and non-Typhimurium predicted notification rates with 95% CI per 100,000 by age group, Australia, 2000–2013. Note: the first five age groups are single years to highlight the pattern of Salmonella in young children.

Of the salmonellosis notifications included in this analysis, 49.7% (61,557/123,762) were in males. In the state and territory model, there was no significant difference between the sexes for infection with non-Typhimurium *Salmonella*, but we found differences for *S*. Typhimurium infection with lower rates in males (Table 1; IRR 0.93; 95% CI 0.91–0.95). The median age at onset of salmonellosis cases included in this study was 22 years old (range <1–108 years). Non-Typhimurium *Salmonella* incidence rates peaked at 0–11 months of age and *S*. Typhimurium incidence rates peaked at 1–2 years of age, with a relative increase in the IRR for *S*. Typhimurium in those 80 years and older (Fig 3). The age distributions of cases by states and territories were consistent with this national age distribution (S2 Fig) although there were differences in the proportion of all salmonellosis that is due to *S*. Typhimurium. *S*. Typhimurium rates were higher than non-Typhimurium rates in most age groups in the south eastern continental states (ACT, NSW, Vic., and SA), while non-Typhimurium *Salmonella* rates were higher in most age groups elsewhere. The two northern-most Australian states (NT and Qld.) had higher rates of infection in 0–4 year old children than other states and territories (S2 Fig).

**Table 1:** Incident rate ratios calculated using negative binomial regression of S. Typhimurium and non-Typhimurium Salmonella by gender, age, state and time, 2000–2013.

	Typhimurium			Non-Typhimurium		
	IRR^a^	95% CI ^b^	P-value	IRR^a^	95% CI^b^	P-value
Sex (reference = female)						
Male	0.93	0.91–0.95	<0.001	1.01	0.98–1.03	0.60
Age groups (reference = <1)						
1	1.28	1.18–1.39	<0.001	0.77	0.72–0.83	<0.001
2	1.04	0.96–1.13	0.35	0.33	0.31–0.36	<0.001
3	0.89	0.82–0.97	0.01	0.19	0.18–0.21	<0.001
4	0.69	0.64–0.76	<0.001	0.13	0.12–0.14	<0.001
5–9	0.41	0.38–0.45	<0.001	0.08	0.07–0.08	<0.001
10–14	0.26	0.24–0.28	<0.001	0.05	0.05–0.06	<0.001
15–19	0.27	0.25–0.29	<0.001	0.06	0.06–0.07	<0.001
20–24	0.33	0.30–0.35	<0.001	0.10	0.09–0.11	<0.001
25–29	0.29	0.27–0.31	<0.001	0.10	0.09–0.10	<0.001
30–34	0.23	0.21–0.24	<0.001	0.08	0.07–0.08	<0.001
35–39	0.17	0.16–0.19	<0.001	0.06	0.05–0.06	<0.001
40–44	0.16	0.14–0.17	<0.001	0.06	0.05–0.06	<0.001
45–49	0.14	0.13–0.16	<0.001	0.07	0.06–0.07	<0.001
50–54	0.15	0.13–0.16	<0.001	0.07	0.06–0.07	<0.001
55–59	0.14	0.13–0.16	<0.001	0.07	0.06–0.07	<0.001
60–64	0.15	0.13–0.16	<0.001	0.08	0.07–0.08	<0.001
65–69	0.16	0.14–0.17	<0.001	0.08	0.07–0.08	<0.001
70–74	0.17	0.15–0.18	<0.001	0.08	0.07–0.09	<0.001
75–79	0.17	0.15–0.19	<0.001	0.08	0.07–0.08	<0.001
80–84	0.20	0.18–0.23	<0.001	0.09	0.08–0.10	<0.001
85+	0.22	0.20–0.24	<0.001	0.08	0.07–0.09	<0.001
Trend over time by state and territory (2000–2013)						
ACT	1.12	1.10–1.14	<0.001	1.03	1.01–1.05	<0.01
NSW	1.07	1.07–1.08	<0.001	1.04	1.04–1.05	<0.001
NT	1.03	1.01–1.05	<0.01	1.02	1.00–1.03	<0.01
QLD	1.04	1.03–1.05	<0.001	0.99	0.98–1.00	0.01
SA	1.05	1.04–1.06	<0.001	1.05	1.04–1.06	<0.001
TAS	1.04	1.02–1.06	<0.001	1.04	1.02–1.05	<0.001
VIC	1.08	1.08–1.09	<0.001	1.06	1.05–1.07	<0.001
WA	1.01	1.00–1.02	0.02	1.04	1.03–1.05	<0.001

^a^Incidence rate ratio

^b^Confidence interval

**Fig 4:**
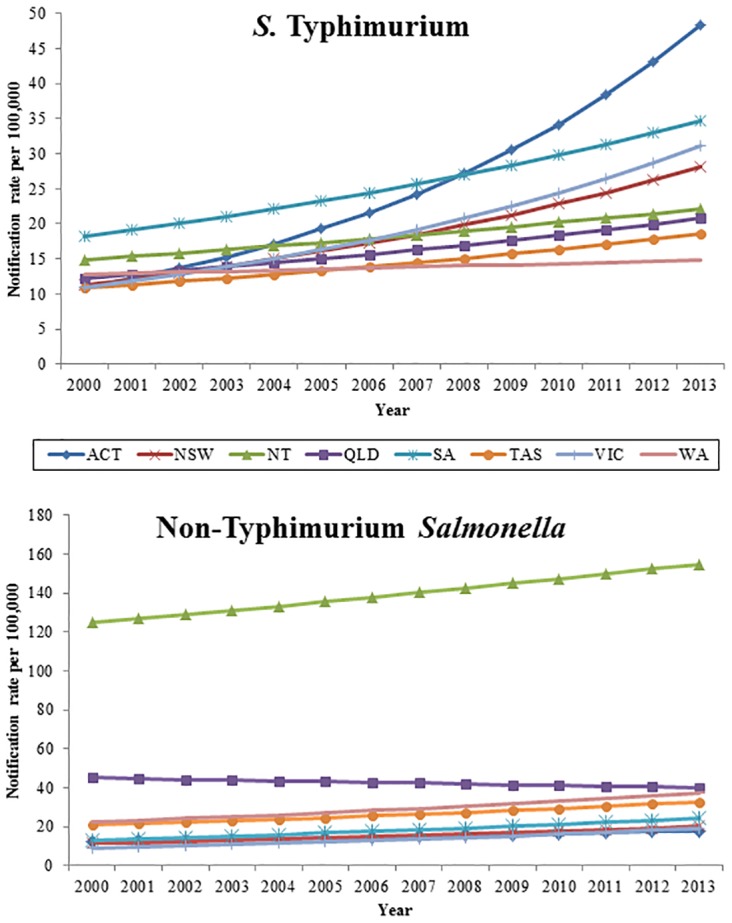
Negative binomial regression margins plot of *S*. Typhimurium and non-Typhimurium predicted notification rates by state and territory, Australia 2000–2013.

At the start of the period, the incidence rate of *S*. Typhimurium was similar across the eastern states and territories but was significantly higher in the western states—WA, NT and SA (Fig 4). Similarly, non-Typhimurium *Salmonella* rates were significantly higher in the northern and western states and territories (NT, Qld, WA, and SA) and in Tasmania than in the south eastern continental states and territories. From 2000 to 2013, the incidence rates of *S*. Typhimurium significantly increased in all states and territories with the highest incidence rate ratios in the ACT (IRR 1.12; 95% CI 1.10–1.14) and Vic. (IRR 1.08; 95% CI 1.08–1.09). During the same time period, incidence rates of non-Typhimurium *Salmonella* significantly increased in all states and territories, except Queensland (IRR 0.99; 95% CI 0.98–1.00). While significant, the regression model for non-Typhimurium *Salmonella* in the NT did not fit the data well, and suggests a more complex pattern over time in this territory (S1 Fig). Trends in ACT, SA, and Tas. remained significant when outlier years were removed from the analysis. Individual state and territory regression lines plotted against the crude rates for both *S*. Typhimurium and non-Typhimurium *Salmonella* can be found in S1 Fig.

## Discussion

Rates of reported salmonellosis have increased significantly in Australia during the last decade and are at unprecedented levels. In particular, *S*. Typhimurium was responsible for over 40% of notifications and is increasing in all states and territories. There is a need for Australian health authorities to identify the key sources of salmonellosis serovars in different states and territories to identify effective ways to substantially reduce infection and improve control of this emerging infection.

The relative importance of *S*. Typhimurium varies by state and territory, with higher rates of non-Typhimurium *Salmonella* in tropical areas. In the Northern Territory, where the majority of people live in a tropical climatic zone, the rate of non-Typhimurium *Salmonella* was about 10 times higher than in most other states and territories. Queensland and Western Australia—the only other states to have regions with tropical climatic zones—had the next highest rates of non-Typhimurium *Salmonella*. Analyzing specific non-Typhimurium serovars in these states and territories over the time period may help to elucidate this pattern and assist with identifying priority serovars for investigation.

The higher incidence of *S*. Typhimurium in the 1–2 year old age group, compared to those under 1, may indicate the importance of foodborne transmission for *S*. Typhimurium. This is consistent with the results of foodborne outbreak investigations, with 92% (56/61) of *Salmonella* foodborne outbreaks attributed to *S*. Typhimurium in Australia in 2011 [7]. Of these, a specific food vehicle was identified in 73% (41/56) outbreaks and 71% (29/41) were associated with the consumption of eggs and egg-based dishes [7]. In addition, over the time period of this study, there has been a significant increase in the number of *Salmonella* outbreaks associated with eggs, with *S*. Typhimurium responsible for nearly all egg-related outbreaks [20]. Of the 5 non-Typhimurium *Salmonella* outbreaks in 2011, 80% (4/5) had a known food vehicle [7]. Poultry was the responsible vehicle in 75% (3/4) of these outbreaks and the remaining outbreak with a known food vehicle was associated with fruit [7]. The higher incidence of non-Typhimurium *Salmonella* in the 0–11 month age group than in any other age group suggests that these serovars may have other transmission pathways, such as environmental, waterborne and zoonotic routes. An Australian expert elicitation estimated that 15% of all *Salmonella* illness is due to environmental sources, 5% is transmitted through contaminated water, and 4% is zoonotic [1]. For example, a case control study of *S*. Mississippi in Tasmania found indirect contact with native animal birds, untreated drinking water and travel within the state as significant predictors of infection [10] and a study in the Northern Territory found a number of non-Typhimuirum *Salmonella* serovars in household environments, including in animal faeces, soil, and vaccum cleaner contents [21].

Unlike in the US, Canada, China, and most of the European Union (EU), *S*. Enteritidis is less common than *S*. Typhimurium in Australia and only makes up a small proportion of notifications (5.7%) [22–25]. The increasing trend of salmonellosis seen in Australian states and territories also differs from the US and European experience. Although there was no change in the overall rate of *Salmonella* in 2012, compared to 1996–1998 and 2006–2008, the US saw a decrease in *S*. Typhimurium during this time period, as well as a decrease in the overall rate of *Salmonella* in 2013 compared to 2010–2012 [22,26]. The EU has shown a significantly decreasing trend of *Salmonella* notifications from 2008–2012. Crude notification rates of salmonellosis are higher in Australia, with a rate of 49.5 per 100,000 in 2012, compared to 22.2 (range 1.8–97.5) cases per 100,000 in the EU in 2012 and 15.9 cases per 100,000 in the US in 2013 [22, 24]. While Canada has seen increases in their overall rate of *Salmonella* between 2003 and 2009, rates were lower (16.3–18 per 100,000) than in Australia and the increase has largely been due to *S*. Enteritidis [23].

There were several limitations to our analysis. We were unable to remove travel associated cases from the data; however a previous study of data from circa 2010 found that approximately 15% of Salmonella notifications were travel associated [13]. Our unit of analysis was state and territory, so we were unable to examine trends at a finer scale or by climatic zones. In addition, we could not distinguish between outbreak and sporadic cases. We could not account for health-seeking behavior or increases in testing, which may have influenced the IRRs of S. Typhimurium and non-Typhimurium *Salmonella*. Methods for further characterization of *S*. Typhimurium isolates vary across states and territories and including Pulse-Field Gel electrophoresis (PFGE), multilocus variable number tandem repeat analysis (MLVA), and phage typing [27–29]. Two percent of all *Salmonella* notifications were monophasic *S*. subspecies I isolates, some of which were classified with *S*. Typhimurium based on their serotyping and phage typing characteristics. If phage typing was not performed this may have resulted in a small number of misclassifications in the likely serovar category. Although the method of testing for *Salmonella* remained relatively consistent over the study time period [30], we were unable to account for any changes that may have occurred in the rate of testing.

In Australia, culture has been the mainstay of clinical diagnostic testing for *Salmonella*; however, there has been increased adoption of culture-independent testing in diagnostic labs. Subsequent to this analysis, in 2014, the first full year that culture-independent diagnostic testing (CIDT) was widely used in Australian diagnostic labs, notification rates of salmonellosis increased by 22% nationally from 2013 to 69.7 notifications per 100,000 [15]. This is the largest recorded annual increase in rate since notifications began and the extent of the impact CIDT had on this rate is unknown. Although CIDT provides quicker results and can be cheaper than culture, an isolate is needed for further characterization [31]. A survey of FoodNet clinical laboratories in the US found that while only a small proportion of laboratories surveyed (1.3%) were using CIDT, a concerning amount of specimens were either not being cultured (60%) or culture did not yield a pathogen (3%) [32]. As whole genome sequencing (WGS), which offers highly discriminatory molecular markers for cluster detection [33], becomes available for routine *Salmonella* typing, it will be important that isolates continue to be cultured and consistent characterization methods are used. CIDT without culture results in a loss of data regarding the *Salmonella* serovar and other typing information, which presents challenges for outbreak identification and monitoring disease burden and trends. Further research on WGS in the Australian setting will help determine whether WGS can contribute to effectively detecting *Salmonella* clusters for investigation.

This study provides important insights into the epidemiology of *Salmonella* infections in Australian states and territories. We observed sustained increases in both *S*. Typhimurium and non-Typhimurium *Salmonella* between 2000 and 2013, with geographic differences in both rates and trends. With the increasing use of CIDT, meaningful comparisons in disease rates over time may become more difficult; however novel typing methods such as whole genome sequencing offers the potential for a richer understanding of salmonellosis in Australia. This improved understanding is needed to inform the control of salmonellosis in Australia.

## Supporting Information

S1 Fig State and territory crude (dots) and predicted (lines with 95% CI) notification rates per 100,000 persons, Australia 2000–2013. (PDF)

S2 Fig *Salmonella* Typhimurium and non-Typhimurium predicted notification rates per 100,000 (with 95% CI) by age group for each State and Territory, 2000–2013. (PDF)

S1 Table Number and proportion of *Salmonella* notifications without serovar data by state and territory, Australia 2000–2013. (DOCX)

S2 Table *Salmonella* spp. cases each year, the proportion of cases excluded due to missing serovar, age or sex data, crude notification rate after exclusions, *S*. Typhimurium notification rate, and Non-Typhimurium notification rate (per 100,000 persons, Australia 2000–2013). (DOCX)

S3 Table Proportion (%) of *S*. Typhimurium and the 20 most notified non-Typhimurium *Salmonella* serovars of total notifications included in this study for each state and territory, Australia, 2000–2013. (DOCX)
