# Supplementary material for: Increasing Incidence of Salmonella in Australia, 2000-2013
Source: PLoS One. 2016 Oct 12;11(10):e0163989. doi: 10.1371/journal.pone.0163989 (PMC5061413; doi:10.1371/journal.pone.0163989)
Supplement: S1 Fig — (PDF) [file pone.0163989.s001.pdf]

S1 Fig. State and territory crude (dots) and predicted (lines with 95% CI) notification rates per 100,000 persons, Australia 2000-2013

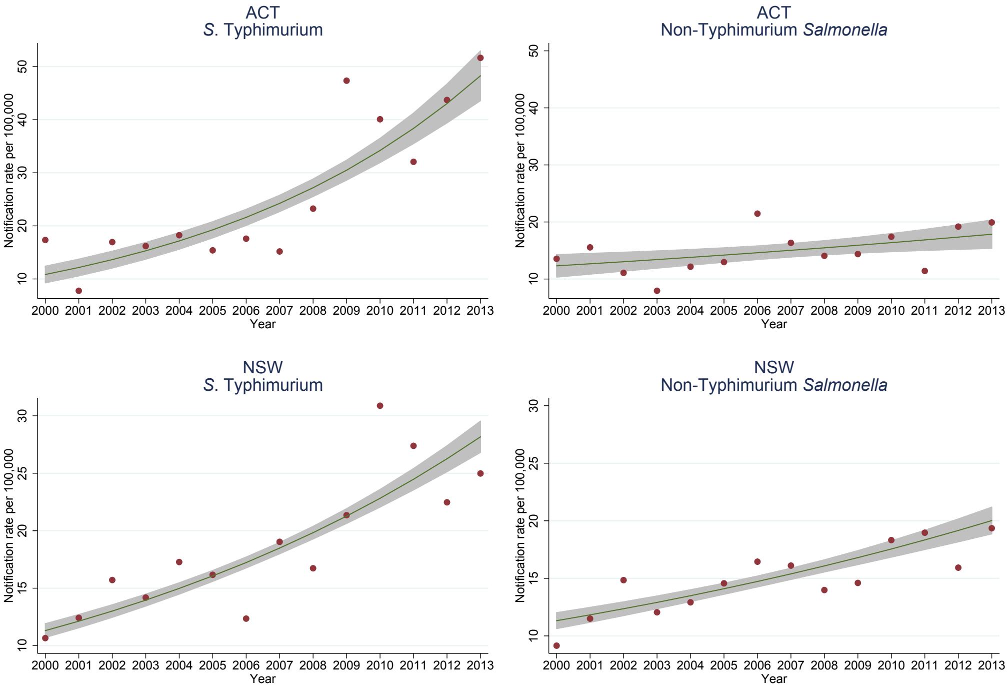

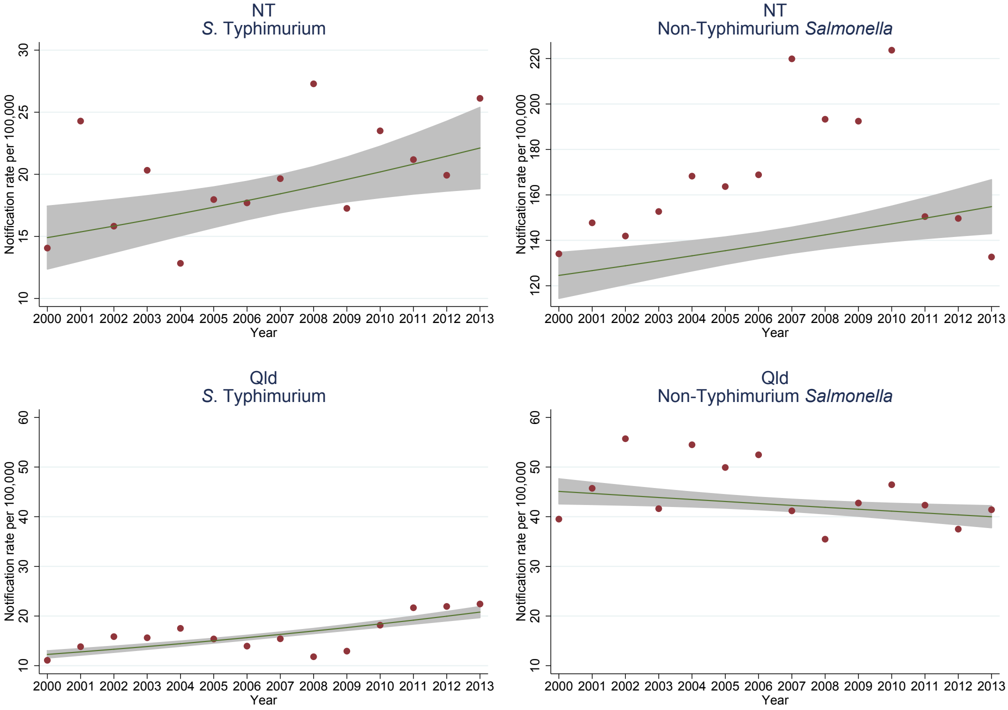
NT
S. Typhimurium
Notification rate per 100,000
Year
NT
Non-Typhimurium Salmonella
Notification rate per 100,000
Year
Qld
S. Typhimurium
Notification rate per 100,000
Year
Qld
Non-Typhimurium Salmonella
Notification rate per 100,000
Year

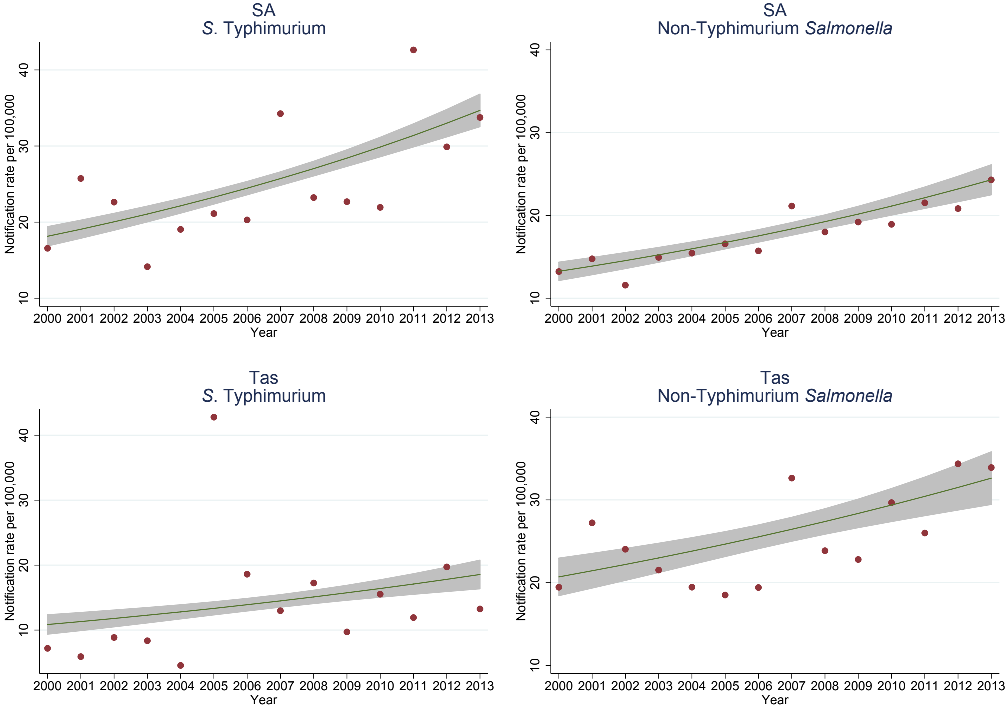
SA
S. Typhimurium
Notification rate per 100,000
10
20
30
40
2000 2001 2002 2003 2004 2005 2006 2007 2008 2009 2010 2011 2012 2013
Year
SA
Non-Typhimurium Salmonella
Notification rate per 100,000
10
20
30
40
2000 2001 2002 2003 2004 2005 2006 2007 2008 2009 2010 2011 2012 2013
Year
Tas
S. Typhimurium
Notification rate per 100,000
10
20
30
40
2000 2001 2002 2003 2004 2005 2006 2007 2008 2009 2010 2011 2012 2013
Year
Tas
Non-Typhimurium Salmonella
Notification rate per 100,000
10
20
30
40
2000 2001 2002 2003 2004 2005 2006 2007 2008 2009 2010 2011 2012 2013
Year

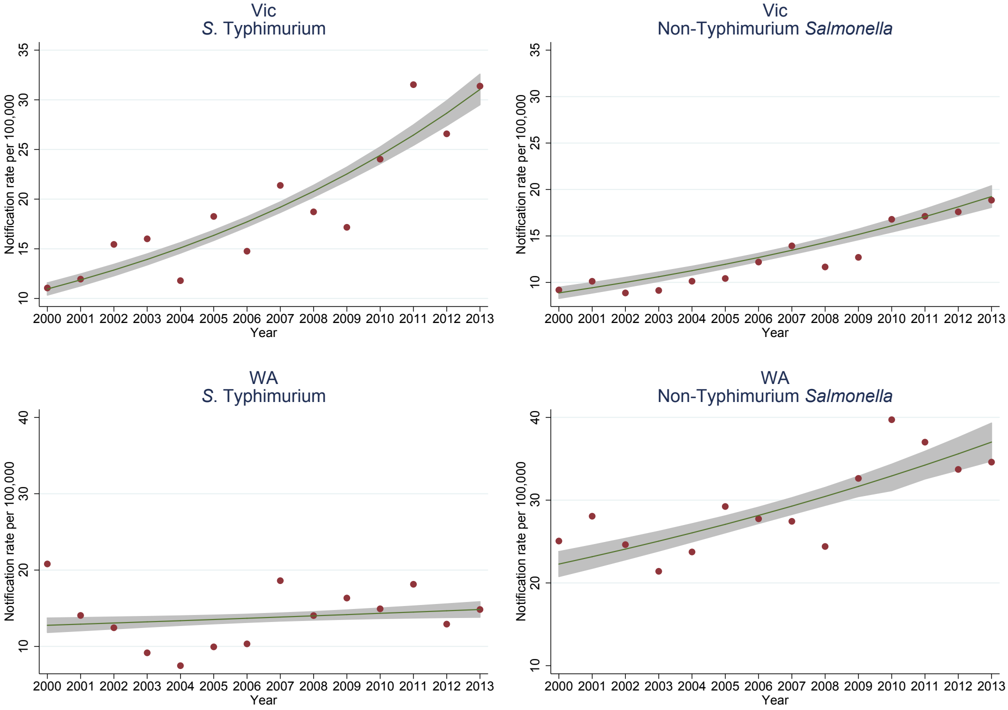
Vic
S. Typhimurium
Vic
Non-Typhimurium Salmonella
WA
S. Typhimurium
WA
Non-Typhimurium Salmonella
Notification rate per 100,000
Year
2000 2001 2002 2003 2004 2005 2006 2007 2008 2009 2010 2011 2012 2013
10 15 20 25 30 35
10 20 30 40
