# Supplementary material for: Increasing Incidence of Salmonella in Australia, 2000-2013
Source: PLoS One. 2016 Oct 12;11(10):e0163989. doi: 10.1371/journal.pone.0163989 (PMC5061413; doi:10.1371/journal.pone.0163989)
Supplement: S2 Fig — (PDF) [file pone.0163989.s002.pdf]

S2 Fig. ***Salmonella* Typhimurium and non-Typhimurium predicted notification rates per 100,000 (with 95% CI) by age group for each State and Territory, 2000-2013**

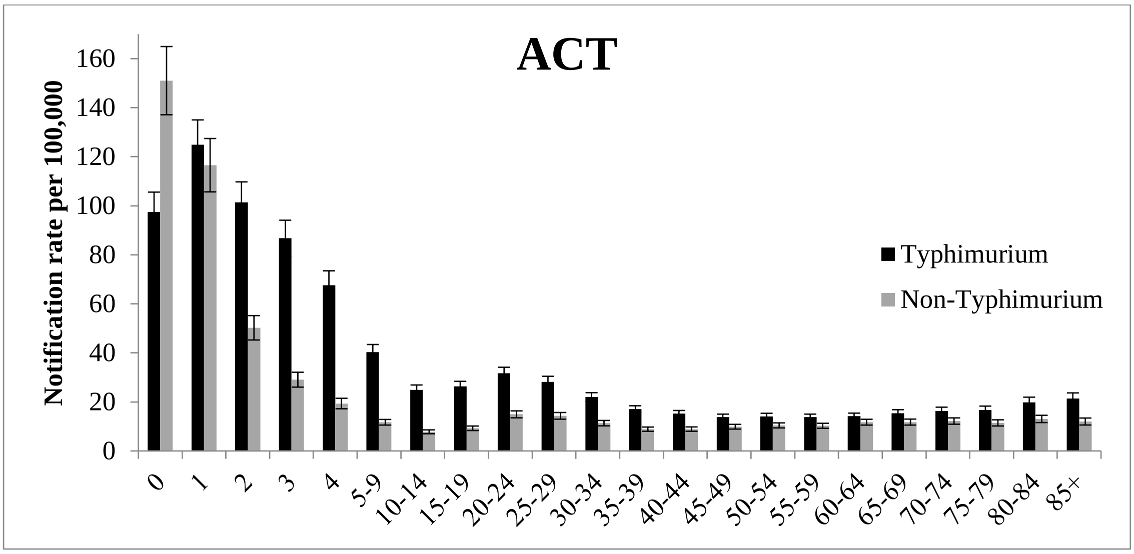

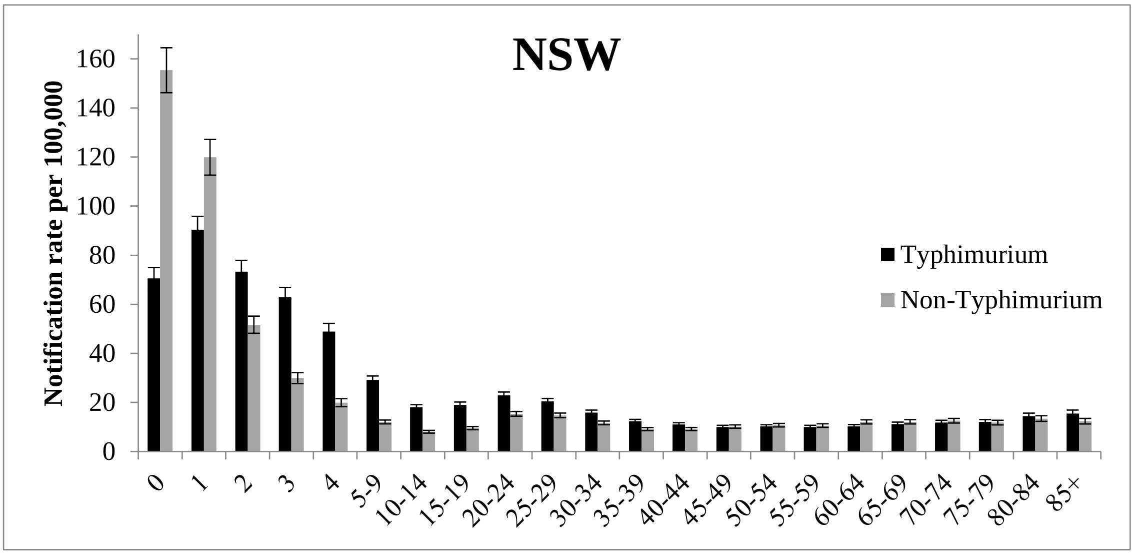

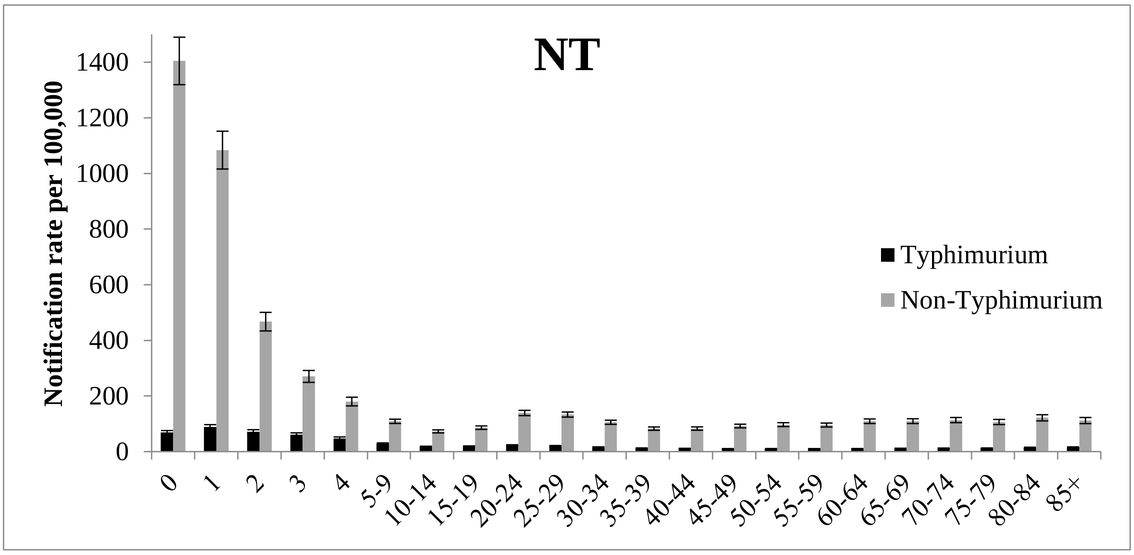

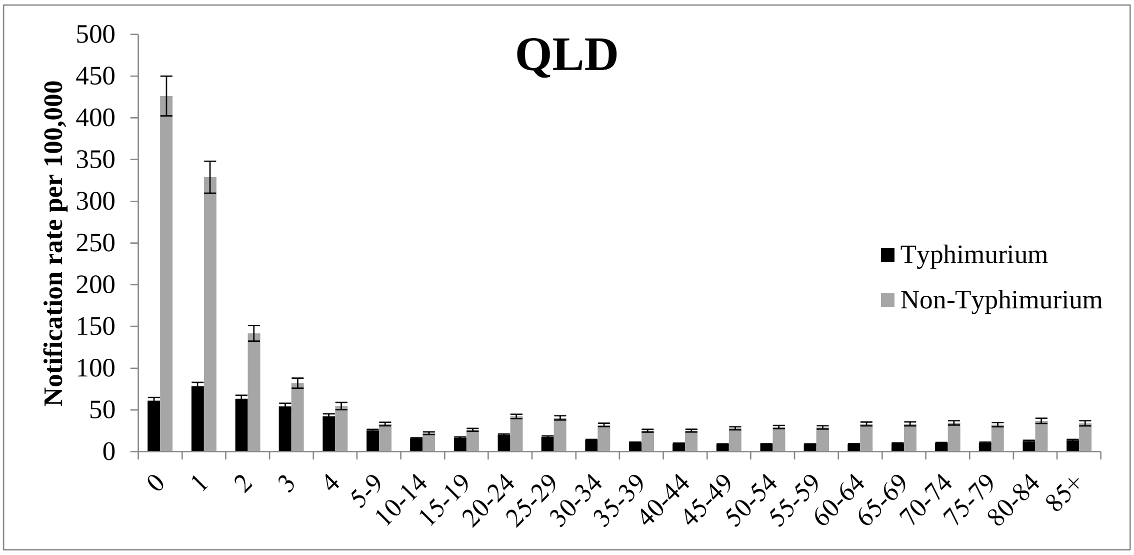
QLD
Notification rate per 100,000
0
50
100
150
200
250
300
350
400
450
500
Typhimurium
Non-Typhimurium
0
1
2
3
4
5-9
10-14
15-19
20-24
25-29
30-34
35-39
40-44
45-49
50-54
55-59
60-64
65-69
70-74
75-79
80-84
85+

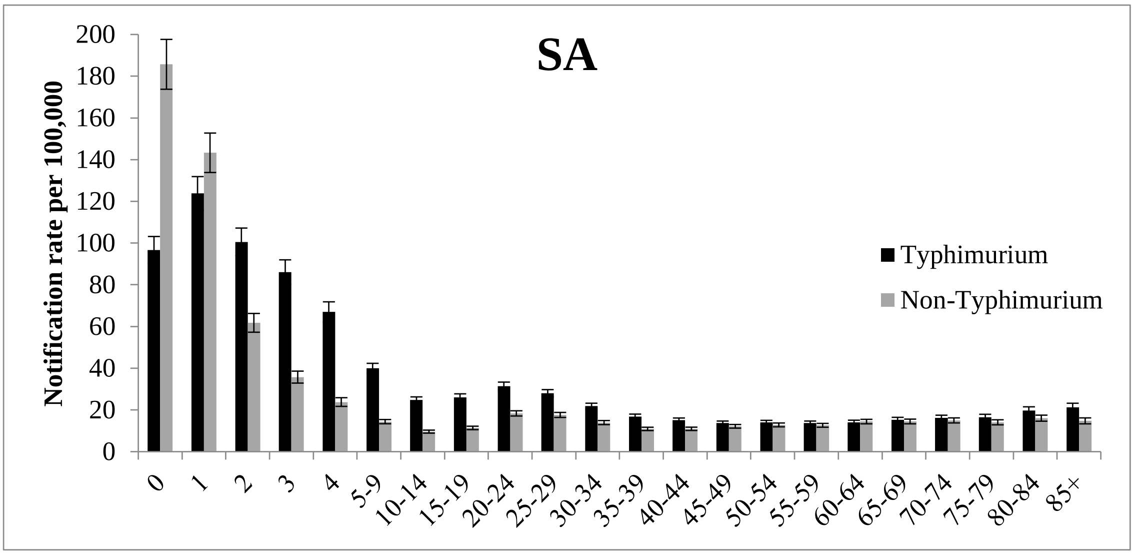
SA
Notification rate per 100,000
0
20
40
60
80
100
120
140
160
180
200
Typhimurium
Non-Typhimurium
0
1
2
3
4
5-9
10-14
15-19
20-24
25-29
30-34
35-39
40-44
45-49
50-54
55-59
60-64
65-69
70-74
75-79
80-84
85+

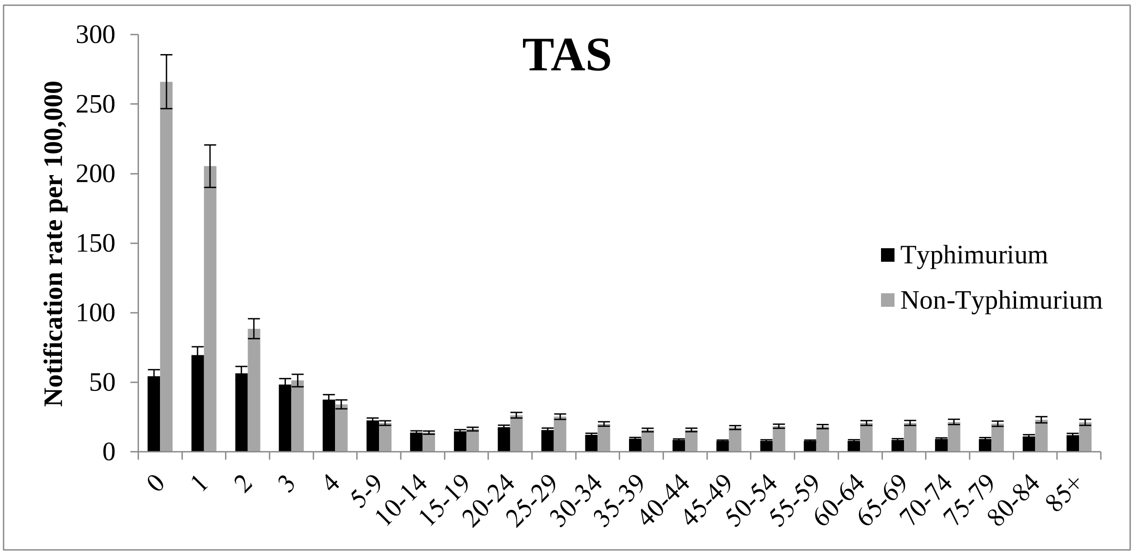
TAS
Notification rate per 100,000
0
50
100
150
200
250
300
Typhimurium
Non-Typhimurium
0
1
2
3
4
5-9
10-14
15-19
20-24
25-29
30-34
35-39
40-44
45-49
50-54
55-59
60-64
65-69
70-74
75-79
80-84
85+

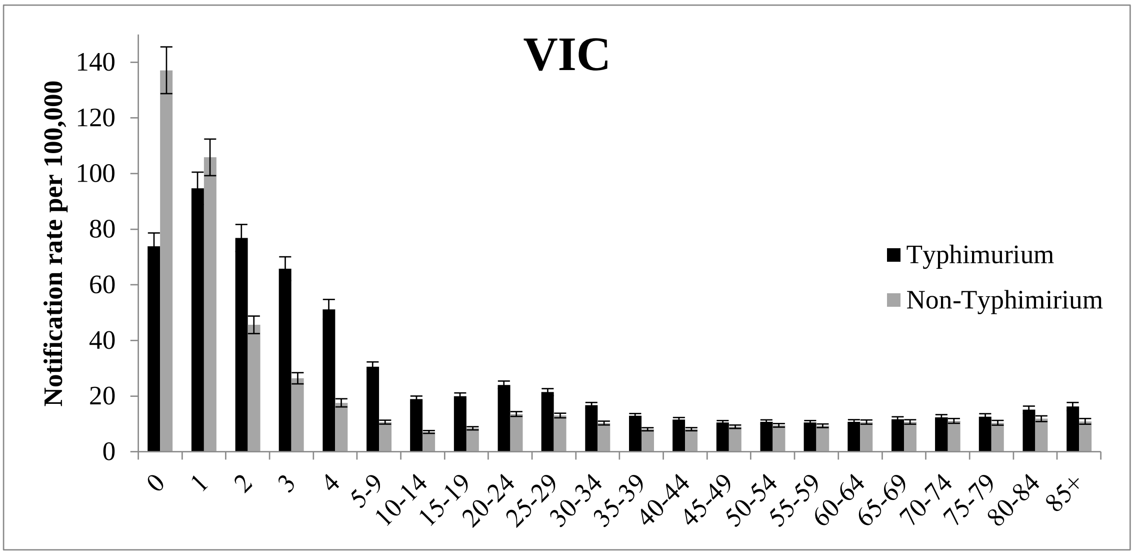
VIC
Notification rate per 100,000
140
120
100
80
60
40
20
0
Typhimurium
Non-Typhimirium
0
1
2
3
4
5-9
10-14
15-19
20-24
25-29
30-34
35-39
40-44
45-49
50-54
55-59
60-64
65-69
70-74
75-79
80-84
85+

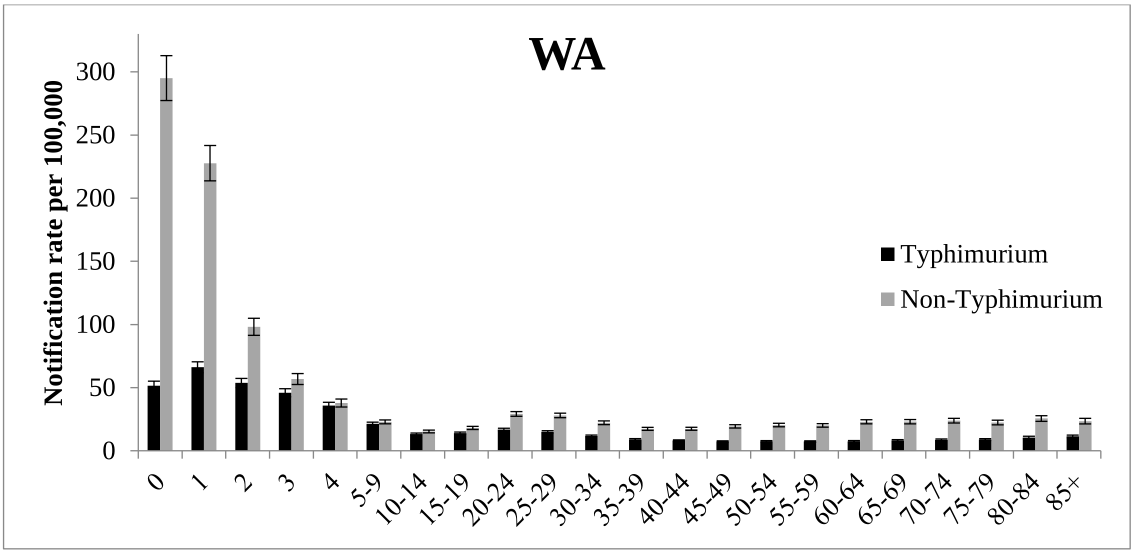
WA
Notification rate per 100,000
300
250
200
150
100
50
0
Typhimurium
Non-Typhimurium
0
1
2
3
4
5-9
10-14
15-19
20-24
25-29
30-34
35-39
40-44
45-49
50-54
55-59
60-64
65-69
70-74
75-79
80-84
85+
