# Supplementary material for: Increasing Incidence of Salmonella in Australia, 2000-2013
Source: PLoS One. 2016 Oct 12;11(10):e0163989. doi: 10.1371/journal.pone.0163989 (PMC5061413; doi:10.1371/journal.pone.0163989)
Supplement: S1 Table — (DOCX) [file pone.0163989.s003.docx]

**S1 Table. Number and proportion of *Salmonella* notifications without serovar data by state and territory, Australia 2000-2013**

| **State** | **Number of notifications without serovar data** | **Total number of notifications** | **Proportion of notifications without serovar data (%)** |
| --- | --- | --- | --- |
| ACT | 23 | 2,028 | 1.1 |
| NSW | 1,355 | 34,100 | 4.0 |
| NT | 189 | 5,850 | 3.2 |
| Qld | 819 | 35,597 | 2.3 |
| SA | 57 | 9,465 | 0.6 |
| Tas. | 49 | 2,772 | 1.8 |
| Vic. | 209 | 24,122 | 0.9 |
| WA | 259 | 13,261 | 2.0 |
| **Total** | 2,960 | 127,195 | 2.3 |
