# Supplementary material for: Increasing Incidence of Salmonella in Australia, 2000-2013
Source: PLoS One. 2016 Oct 12;11(10):e0163989. doi: 10.1371/journal.pone.0163989 (PMC5061413; doi:10.1371/journal.pone.0163989)
Supplement: S2 Table — (DOCX) [file pone.0163989.s004.docx]

**S2 Table.** ***Salmonella* spp. cases each year, the proportion of cases excluded due to missing serovar, age or sex data, crude notification rate after exclusions, *S.* Typhimurium notification rate, and Non-Typhimurium notification rate (per 100,000 persons, Australia 2000–2013).**

| **Year** | **Total cases (n)** | **Proportion excluded (%)** | **Crude rate**  **(per 100,000 persons)** | ***S*. Typhimurium rate (per 100,000 persons)** | **Non-Typhimurium rate (per 100,000 persons)** |
| --- | --- | --- | --- | --- | --- |
| 2000 | 6,154 | 5.2 | 30.6 | 12.4 | 18.3 |
| 2001 | 6,995 | 3.8 | 34.9 | 13.7 | 21.3 |
| 2002 | 7,824 | 2.9 | 39.0 | 15.7 | 23.2 |
| 2003 | 6,948 | 3.4 | 34.1 | 14.4 | 19.7 |
| 2004 | 7,752 | 2.3 | 38.0 | 14.8 | 23.2 |
| 2005 | 8,346 | 2.0 | 40.5 | 16.9 | 23.6 |
| 2006 | 8,168 | 1.9 | 39.2 | 14.0 | 25.2 |
| 2007 | 9,398 | 1.7 | 44.3 | 19.8 | 24.5 |
| 2008 | 8,234 | 2.4 | 37.8 | 16.7 | 21.1 |
| 2009 | 9,443 | 2.8 | 42.3 | 18.3 | 24.0 |
| 2010 | 11,828 | 2.7 | 52.3 | 24.0 | 28.2 |
| 2011 | 12,209 | 1.7 | 53.7 | 27.1 | 26.7 |
| 2012 | 11,178 | 2.5 | 48.0 | 23.2 | 24.8 |
| 2013 | 12,718 | 3.6 | 53.0 | 25.8 | 27.2 |
