# Supplementary material for: Increasing Incidence of Salmonella in Australia, 2000-2013
Source: PLoS One. 2016 Oct 12;11(10):e0163989. doi: 10.1371/journal.pone.0163989 (PMC5061413; doi:10.1371/journal.pone.0163989)
Supplement: S3 Table — (DOCX) [file pone.0163989.s005.docx]

**S3 Table. Proportion (%) of *S.* Typhimurium and the 20 most notified non-Typhimurium *Salmonella* serovars of total notifications included in this study for each state and territory, Australia, 2000-2013.**

|  | **ACT** | **NSW** | **NT** | **Qld** | **SA** | **Tas.** | **Vic.** | **WA** |
| --- | --- | --- | --- | --- | --- | --- | --- | --- |
| Typhimurium | 64.1 | 55.7 | 10.7 | 27.0 | 58.6 | 35.8 | 60.3 | 32.0 |
| Enteritidis | 4.7 | 4.4 | 2.0 | 4.1 | 5.0 | 3.6 | 5.7 | 15.5 |
| Virchow | 3.0 | 3.5 | 5.9 | 11.6 | 2.2 | 1.7 | 3.0 | 1.4 |
| Saintpaul | 1.8 | 1.9 | 9.3 | 8.4 | 1.9 | 1.2 | 1.7 | 4.6 |
| Birkenhead | 0.5 | 3.4 | <0.1 | 4.9 | 0.1 | <0.1 | 0.4 | 0.1 |
| Infantis | 2.0 | 2.7 | 2.1 | 0.7 | 3.9 | 1.1 | 2.6 | 2.0 |
| Paratyphi B bv Java | 1.9 | 2.1 | 2.4 | 1.4 | 1.4 | 1.0 | 1.9 | 3.4 |
| Chester | 0.6 | 1.2 | 3.2 | 2.7 | 2.2 | 0.6 | 0.7 | 2.9 |
| Muenchen | 0.8 | 0.9 | 2.8 | 2.2 | 1.4 | 0.3 | 0.5 | 2.9 |
| Bovismorbificans | 1.6 | 2.1 | 0.5 | 0.9 | 2.3 | 0.9 | 1.5 | 1.1 |
| Aberdeen | 0.1 | 0.3 | 1.3 | 4.1 | 0.1 | 0.2 | 0.3 | 0.1 |
| Hvittingfoss | 0.6 | 0.5 | 1.1 | 3.3 | 0.2 | 0.1 | 0.6 | 0.4 |
| Stanley | 1.7 | 1.3 | 0.3 | 0.7 | 1.0 | 1.2 | 1.8 | 1.7 |
| Mississippi | 0.3 | 0.2 | 0.1 | 0.2 | 0.2 | 41.9 | 0.5 | 0.1 |
| Waycross | 0.1 | 1.0 | 0.1 | 2.8 | 0.1 | <0.1 | 0.1 | <0.1 |
| Weltevreden | 0.7 | 0.6 | 3.3 | 1.2 | 0.5 | 0.3 | 0.7 | 1.1 |
| Anatum | 0.2 | 0.4 | 2.7 | 1.3 | 1.0 | 0.2 | 0.4 | 1.6 |
| Agona | 0.8 | 1.0 | 0.6 | 0.9 | 1.0 | 0.6 | 0.8 | 0.8 |
| Newport | 0.8 | 0.7 | 0.3 | 0.4 | 1.1 | 1.1 | 1.5 | 1.1 |
| Singapore | 0.5 | 1.3 | 0.2 | 0.4 | 0.8 | 0.2 | 0.6 | 1.2 |
| Potsdam | 0.6 | 0.6 | 0.4 | 1.1 | 0.6 | 0.7 | 0.3 | 0.6 |
